# Supplementary figures and images for: Targeted Metabolome and Transcriptome Analyses Reveal the Pigmentation Mechanism of Hippophae (Sea Buckthorn) Fruit
Source: Foods. 2022 Oct 20;11(20):3278. doi: 10.3390/foods11203278 (PMC9602349; doi:10.3390/foods11203278)

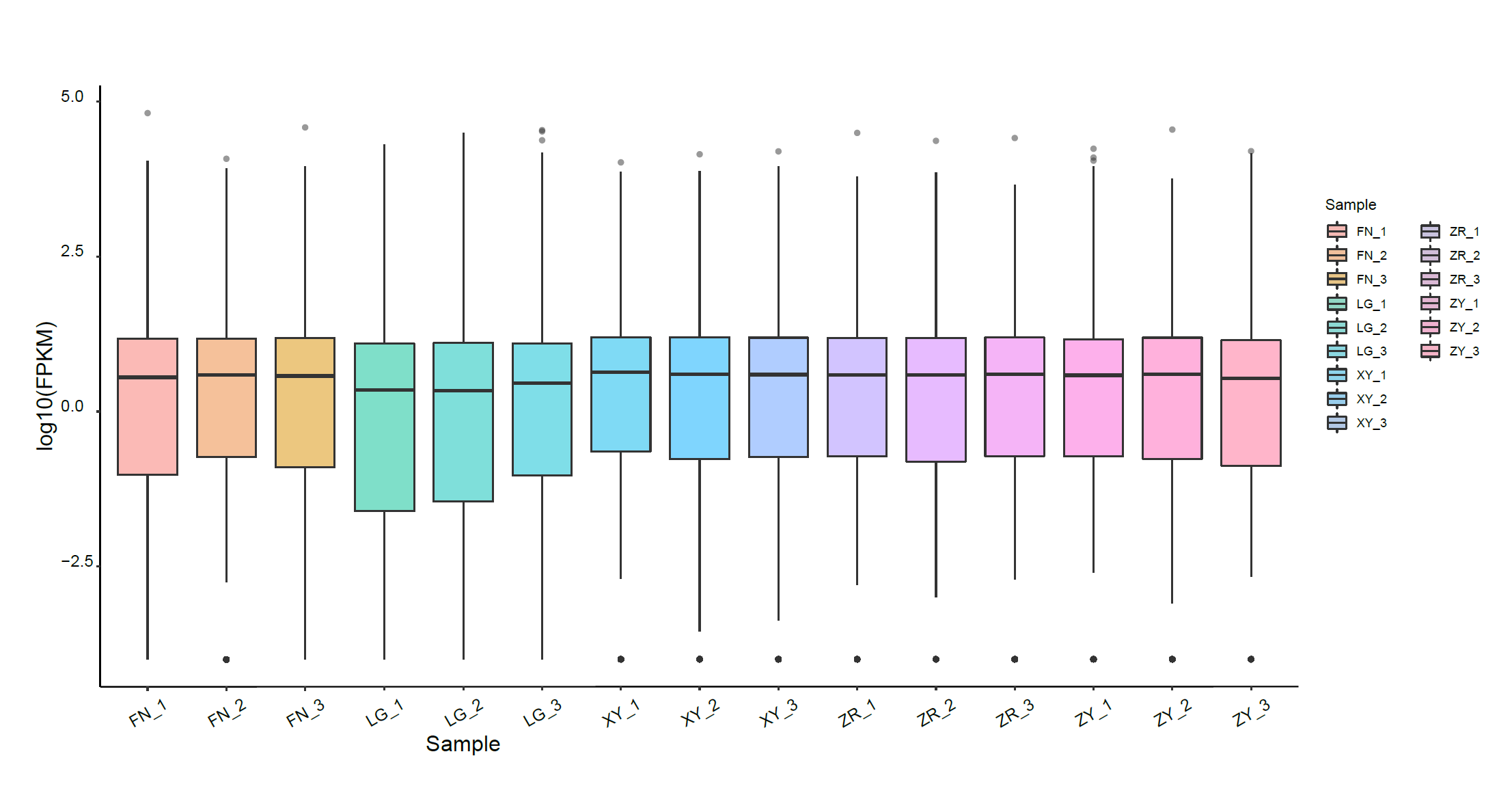

Supplement: Supplementary file 1 [file foods-11-03278-s001.zip › Supplementary Figure S1 Gene expression distribution boxplot.tif]

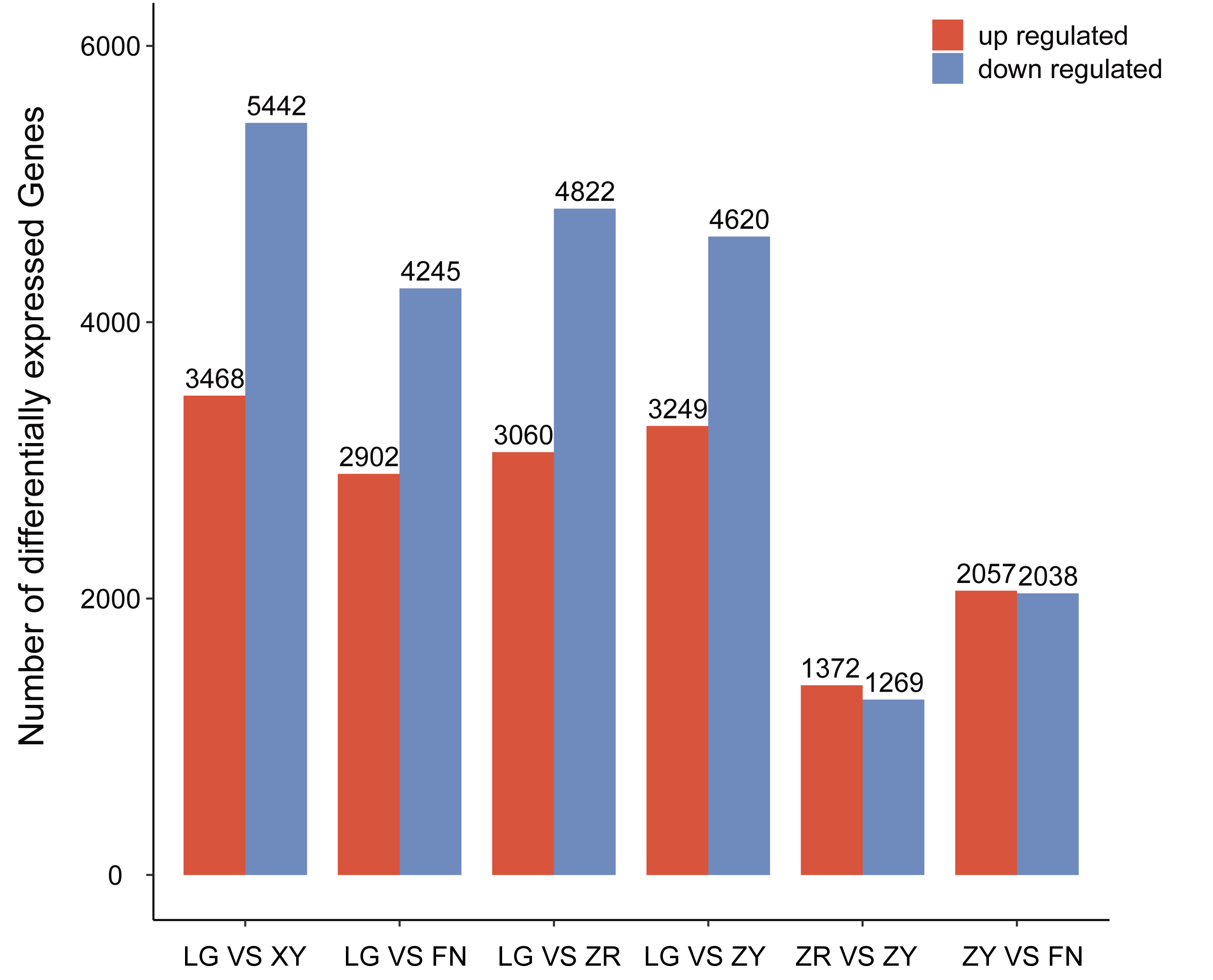

Supplement: Supplementary file 1 [file foods-11-03278-s001.zip › Supplementary Figure S2 Number of DEGs between sample groups.tif]

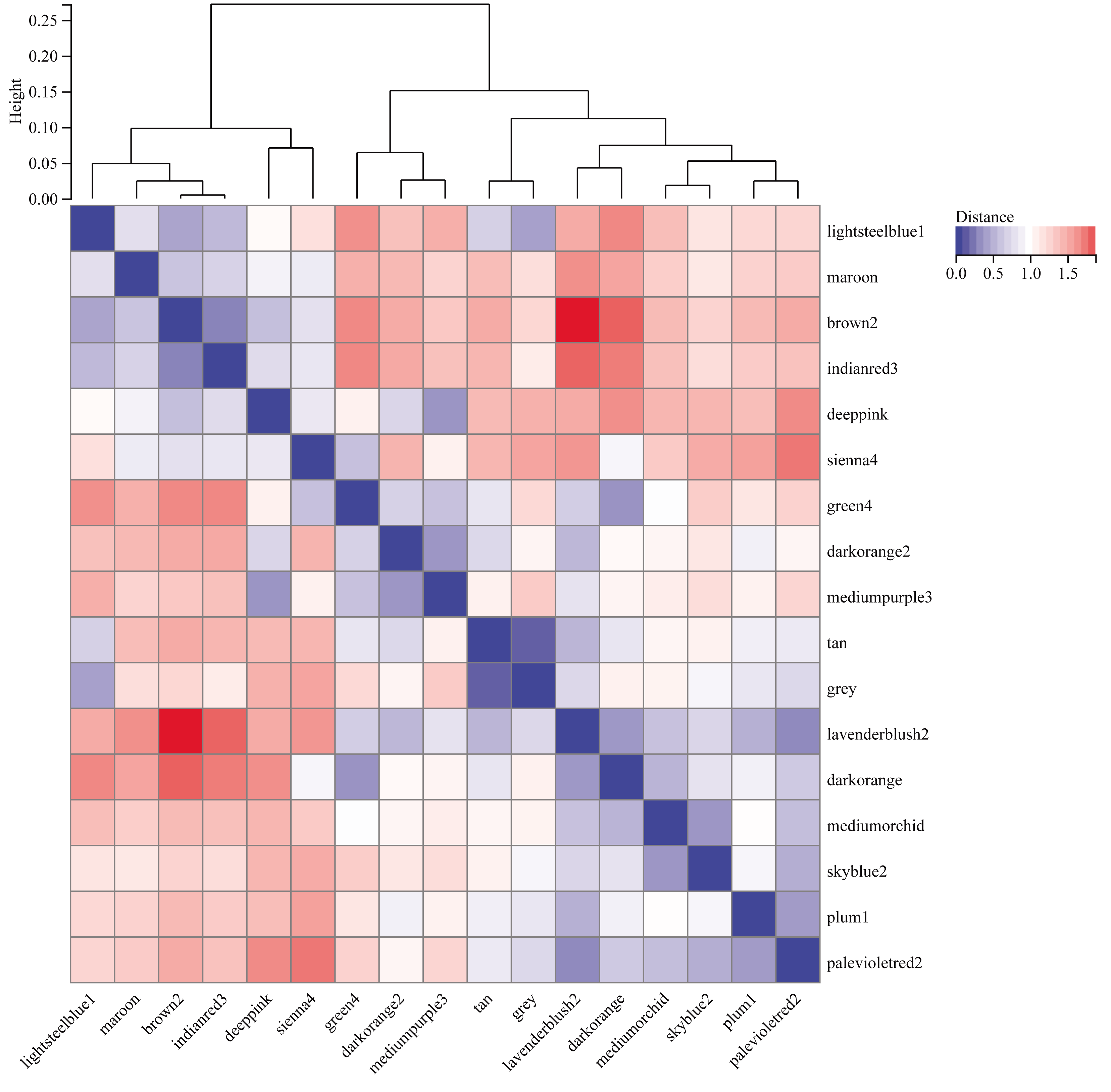

Supplement: Supplementary file 1 [file foods-11-03278-s001.zip › Supplementary Figure S3 Relationships among the modules.tif]

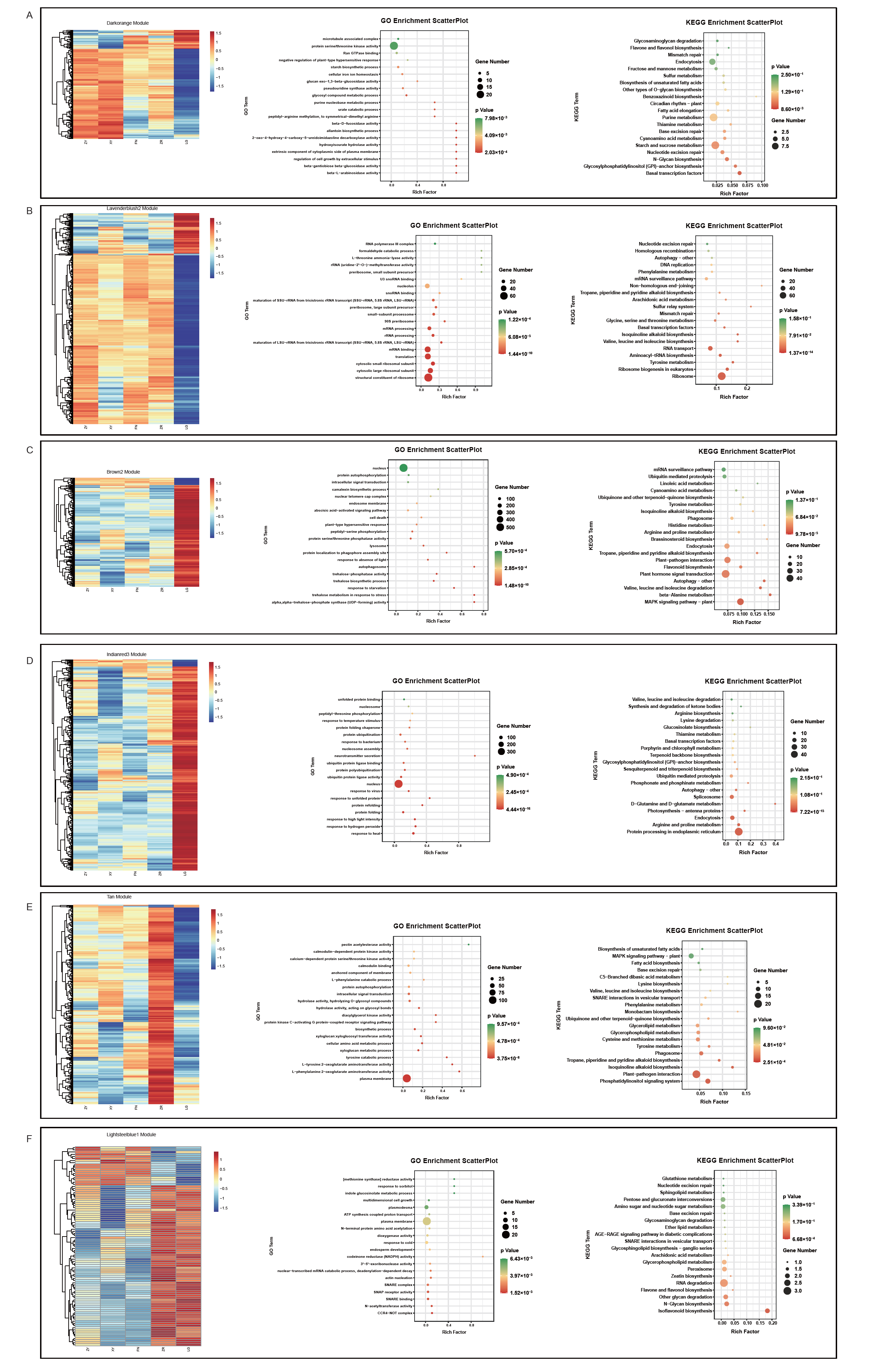

Supplement: Supplementary file 1 [file foods-11-03278-s001.zip › Supplementary Figure S4 Heatmap of FPKM, and GO and KEGG scatterplot of module genes.tif]
